# AI–enabled comprehensive patient safety management in acupuncture: from risk identification to continuous quality improvement

**DOI:** 10.3389/fmed.2026.1959255

**Published:** 2026-08-28

**Authors:** Xixi Wei, Chao Niu

**Affiliations:** 1 Heji Hospital Affiliated to Changzhi Medical College, Changzhi, China; 2 Changzhi Hospital of Traditional Chinese Medicine, Changzhi, China

**Keywords:** acupuncture, artificial intelligence, digital health, human–AI collaboration, patient safety, risk management

## Abstract

Acupuncture, as an important component of traditional medicine, has been widely integrated into the management of various clinical conditions. Although acupuncture is generally considered safe and well tolerated, adverse events, including bleeding, pneumothorax, neurovascular injury, infection, and retained needles, may still occur. Current safety management approaches largely rely on operator experience and manual monitoring, which may be insufficient to address individual patient variability and dynamic procedural risks. In recent years, Artificial Intelligence (AI) has emerged as a promising technological approach to enhance patient safety management in acupuncture practice. This review focuses on the entire acupuncture care pathway and summarizes the potential applications of AI in risk identification, intelligent assistance, and safety monitoring. Current evidence suggests that AI technologies may support high-risk patient screening, personalized treatment planning, real-time procedural monitoring, and safety data management. However, existing evidence remains largely limited to technology development and preliminary validation, with insufficient clinical evidence. Future efforts should focus on establishing a human–AI collaborative framework to develop comprehensive intelligent patient safety management systems, facilitating the transition of acupuncture safety management from experience-driven practices toward data-informed decision-making and proactive risk prevention.

## 1 Introduction

Acupuncture has been widely applied in the management of pain-related disorders, neurological diseases, disorders of gut–brain interaction, and supportive care in oncology. Evidence mapping studies indicate that systematic reviews of acupuncture have covered dozens of adult health conditions, reflecting the continued expansion of its clinical applications (1). With the accumulation of randomized controlled trials and evidence-based research, acupuncture has gradually been incorporated into several international guidelines and expert consensus statements for conditions such as chronic musculoskeletal pain and integrative oncology care (2, 3). However, compared with efficacy evaluation, acupuncture safety and the establishment of comprehensive safety management systems have received relatively limited attention. Common adverse events include needling-related pain, minor bleeding, subcutaneous bruising, dizziness, and vasovagal reactions, whereas more serious events, including pneumothorax, neurovascular or organ injury, infection, needle breakage, and retained needles, have also been reported (4, 5). Moreover, inconsistencies in the definitions of adverse events, needling locations, insertion depths, and causal assessment methods have hindered the systematic identification of risk factors (6). Severe adverse events are often associated with multiple factors, including patient-specific conditions and anatomical variations, operator experience, acupuncture parameters, aseptic practices, and clinical workflow. Importantly, some of these risks may be preventable through optimized management strategies (7). Therefore, improving the precision, traceability, and risk management capabilities of acupuncture practice has become an essential component of its modernization.

Currently, acupuncture safety management remains largely dependent on practitioners’ anatomical knowledge, clinical experience, and real-time judgment. Key aspects of acupuncture care, including patient risk assessment, acupoint localization, parameter control, recognition of abnormal responses, needle inventory verification, and post-treatment follow-up, still lack continuous, objective, and quantitative support. Artificial Intelligence (AI), through advances in computer vision, deep learning, multimodal analysis, intelligent sensing, and robotics, provides new opportunities to enhance the objectivity, standardization, and personalization of acupuncture practice (8). Existing studies have explored AI-assisted acupoint localization, anatomical recognition using medical imaging or ultrasound, as well as needle counting and procedural status monitoring (9–11). These technologies have the potential to reduce localization errors, assist in controlling procedural parameters, and minimize manual verification errors. However, most current applications remain at the stage of prototype development or small-scale validation, with limited evidence from real-world clinical settings.

Recent reviews have summarized the applications of AI in acupuncture from perspectives including precision integrative medicine, algorithm development, and clinical tasks, primarily focusing on acupoint localization, manipulation recognition, disease classification, treatment response prediction, therapeutic monitoring, and clinical decision support (12, 13). Nie et al. categorized AI applications in acupuncture into acupoint selection, acupoint localization, safety enhancement, and efficacy prediction, while also incorporating needle counting and acupuncture process monitoring (14). Recommendations from acupuncture research organizations have further highlighted the importance of data standardization, clinical documentation, digital literacy, privacy protection, ethical governance, and interdisciplinary collaboration in advancing AI applications (15). However, previous reviews have generally been technology-centered, with safety considered as only one potential application area. A comprehensive framework focusing on patient harm prevention and integrating risk identification, procedural control, adverse event monitoring, and continuous quality improvement remains lacking.

To address these knowledge gaps, this review adopts patient safety management as the central framework and systematically discusses the role of AI across the entire acupuncture care pathway, including pre-procedural risk identification and individualized planning, intra-procedural intelligent guidance and real-time monitoring, post-procedural adverse event recognition, and continuous quality improvement. We extend the concept of acupuncture safety beyond isolated technical procedures toward a comprehensive management system involving patients, practitioners, devices, and clinical workflows. Furthermore, we propose a closed-loop framework integrating risk prediction, precision control, real-time alerting, event documentation, and feedback-driven improvement. Finally, we discuss key translational challenges, including data standardization, model generalizability, clinical validation, human–AI collaboration, algorithm interpretability, ethical responsibility, and regulatory oversight, providing new perspectives for the development of AI-driven comprehensive safety management systems in acupuncture.

## 2 Safety risks in acupuncture and the need for intelligent management

### 2.1 Acupuncture-related safety risks and their underlying determinants

Acupuncture is generally considered a safe and well-tolerated non-pharmacological intervention; however, it is not entirely free of risks. Prospective studies and systematic reviews have shown that common adverse events include needling-related pain, minor bleeding, subcutaneous bruising, hematoma, dizziness, vasovagal reactions, and transient symptom exacerbation, most of which are mild and self-limiting (16, 17). Nevertheless, more serious events, including needle breakage, needle retention, infection, neurovascular injury, and pneumothorax, may still occur. In rare cases, these complications can result in hospitalization, permanent functional impairment, or life-threatening consequences. Therefore, acupuncture safety management should not focus solely on the incidence of severe adverse events but should also consider the cumulative burden of minor events, delayed injuries, and latent risks throughout the clinical workflow.

Acupuncture-related risks are typically determined by the combined effects of patient characteristics, underlying medical conditions, and procedural factors. Previous studies have suggested that chronic pulmonary diseases and a history of thoracic surgery may increase the risk of pneumothorax following acupuncture (18). Anticoagulant or antiplatelet therapy, liver cirrhosis, diabetes, and coagulation disorders may increase bleeding susceptibility (19). Meanwhile, diabetes, chronic kidney disease, cerebrovascular disorders, and pre-existing neurological conditions may influence the risk of nerve-related complications (20). These findings indicate that identical acupoints and procedural parameters may not represent equivalent safety margins across different individuals. Comprehensive assessment incorporating patient health status, medication use, and local anatomical characteristics is therefore essential for individualized risk stratification.

Moreover, acupuncture procedures remain highly dependent on practitioner expertise. Acupoint localization, needle direction, insertion angle, and depth are influenced by anatomical knowledge, clinical experience, and tactile feedback. This dependence becomes particularly important when treating regions such as the chest, back, and neck, where critical neurovascular structures or internal organs may be located near the needling pathway. Conventional standardized parameters may not fully reflect patient-specific anatomical variations. In addition, reports of serious adverse events often lack detailed information regarding needling sites, insertion depths, and operator characteristics, limiting the identification of risk factors and the development of effective prevention strategies (21). Therefore, acupuncture safety should not be regarded merely as a technical issue related to needle manipulation, but rather as a comprehensive risk-control process involving patient screening, treatment planning, procedural implementation, and post-treatment monitoring.

### 2.2 Limitations of conventional safety management and the need for AI integration

Currently, acupuncture safety management relies primarily on standardized training, anatomical education, clinical guidelines, practitioner experience, and manual verification. Although these approaches play essential roles in maintaining basic procedural quality, they remain insufficient for fully addressing individual patient variability and dynamic risks during treatment. Traditional acupoint localization mainly depends on surface anatomical landmarks and proportional measurement methods. However, the underlying tissue structures, neurovascular distribution, and safe insertion ranges associated with specific acupoints may vary according to age, body habitus, posture, and individual anatomical differences. Therefore, acupoints should not be considered merely fixed surface coordinates; instead, acupuncture trajectories and safety margins should be determined based on individualized anatomical information (22).

Furthermore, conventional safety management has limitations in continuous procedural documentation, real-time feedback, and long-term safety surveillance. Parameters such as needle angle, depth, insertion velocity, tissue resistance, and stimulation intensity are rarely recorded continuously, and practitioners typically rely on experience and immediate patient feedback to assess procedural status. Mild or delayed adverse events may also be underreported because monitoring often depends on active patient disclosure. In addition, inconsistencies remain among healthcare institutions regarding adverse event definitions, severity grading systems, and causality assessment, restricting the accumulation of standardized safety data and the development of reliable risk prediction models (23, 24).

The rapid advancement of AI provides new opportunities to overcome these limitations. By integrating patient characteristics, medical imaging, anatomical information, procedural parameters, and post-treatment feedback, AI may facilitate improved risk identification, real-time monitoring, and safety-oriented feedback. However, the current role of AI in acupuncture should not be viewed as a replacement for clinicians or an autonomous decision-making system. Rather, AI should function as a supportive technology that complements clinical expertise and professional judgment by providing objective, continuous, and traceable information. Such integration may promote the transition of acupuncture safety management from experience-driven practices and reactive responses toward data-informed decision-making and proactive risk prevention.

## 3 AI–enabled comprehensive patient safety management across the acupuncture care pathway

Safety risks associated with acupuncture may arise throughout multiple stages of care, including patient screening, treatment planning, needle insertion, needle removal verification, and post-treatment follow-up. In recent years, AI applications in acupuncture have expanded to diverse scenarios, including risk stratification, anatomical assessment, real-time procedural monitoring, needle management, and safety data analytics. However, the safety value of AI should not be evaluated solely based on algorithmic accuracy or technical performance. More importantly, its clinical relevance should be assessed by whether it improves risk identification, reduces the time required to detect abnormalities, and ultimately decreases preventable adverse events. Based on the acupuncture care pathway, this review categorizes AI applications into three stages: pre-procedural, intra-procedural, and post-procedural management, and discusses their potential safety benefits, current evidence, and major limitations (Table 1).

**Table 1:** Artificial intelligence applications across the acupuncture care pathway for comprehensive patient safety management.

Treatment stage	Safety management objective	Major clinical risks	AI application	Key technologies	Potential safety benefits	Current evidence status	Major limitations	References
Pre-procedural	High-risk patient identification	Insufficient identification of risk factors associated with bleeding, pneumothorax, and nerve injury	AI-assisted risk prediction	EHR, real-world data, machine learning, NLP	Integrates comorbidities, medication history, and previous treatment information to support high-risk patient identification and individualized risk stratification	Real-world acupuncture studies combined with AI research from related medical fields	Low incidence of severe adverse events with substantial class imbalance; critical parameters such as needling site, depth, and manipulation technique are rarely recorded in structured formats; outcome definitions remain inconsistent	(18–20)
	Individualized acupoint localization	Variations in surface anatomical landmarks and errors associated with manual localization	AI-assisted acupoint recognition	Computer vision, human keypoint detection, deep learning, anatomy-constrained models	Improves consistency of acupoint localization and provides technical support for reducing localization-related procedural risks	Algorithm development and single-center validation studies	Mostly based on self-established single-center datasets; limited diversity in body habitus, skin characteristics, and age groups; insufficient generalizability under complex postures, occlusion, and soft tissue deformation; clinical safety benefits remain unproven	(25–27)
	Individualized safety boundary assessment	Injury to nerves, vessels, pleura, or other deep structures due to inappropriate insertion depth or direction	AI-assisted anatomical assessment and safety boundary estimation	Ultrasound, CT/MRI, 3D reconstruction, image segmentation	Identifies deep anatomical structures at risk and provides objective guidance for individualized insertion depth and direction	Anatomical/imaging studies combined with related puncture procedure research	Direct evidence in acupuncture remains limited; imaging results are influenced by equipment, operator expertise, and patient positioning; cost, examination time, and training requirements restrict routine application	(18, 28, 29)
	Individualized needle trajectory planning	Mismatch between virtual acupoint coordinates and patient-specific surface and deep anatomical structures	AR-assisted localization and digital anatomical registration	3D reconstruction, spatial registration, AR visualization, motion tracking	Improves visualization of surface localization, needle direction, and procedural pathways to support treatment planning	Prototype development and localization validation studies	Dynamic digital twin systems have not yet been achieved; limited integration of critical deep structures such as nerves, vessels, and pleura; lack of clinical safety outcome validation	(30–32)
Intra-procedural	Dynamic acupoint localization	Acupoint displacement caused by changes in posture, respiration, or soft tissue deformation	AI-assisted real-time acupoint tracking	Real-time computer vision, pose estimation, human keypoint tracking	Dynamically identifies acupoint shifts and provides objective references for procedural adjustment	Algorithm studies and controlled-environment validation	Lack of clinical closed-loop correction studies during acupuncture procedures; recognition performance may be affected by operator hands, needles, and clothing occlusion; soft tissue deformation remains difficult to quantify accurately	(25, 27, 33)
	Protection of deep anatomical structures	Needle tip penetration into nerves, blood vessels, pleura, or internal organs	AI-assisted ultrasound navigation	Ultrasound, image segmentation, needle tracking, spatial registration, robotic navigation	Provides dynamic visualization of the spatial relationship between the needle, needle tip, and vulnerable anatomical structures, potentially reducing blind insertion risks	Small-scale acupuncture studies combined with interventional procedure research	Evidence from related fields cannot directly demonstrate acupuncture safety benefits; thin acupuncture needles remain challenging for ultrasound visualization and tip tracking; high equipment and training requirements	(18, 34, 35)
	Quantification and assisted control of procedural parameters	Lack of objective recording of needle angle, depth, velocity, frequency, and applied force; substantial operator-dependent variability	Sensor-equipped needles and robotic-assisted acupuncture	Force/torque sensors, motion capture, video recognition, robotic control	Improves quantification and reproducibility of acupuncture parameters and supports abnormal procedure detection and control	Engineering validation and animal studies	Mainly limited to prototypes, simulations, or animal experiments; unable to fully replicate clinicians’ tactile feedback and tissue resistance assessment; clinical safety redundancy and emergency stop mechanisms require further validation	(36, 37)
	Detection of abnormal physiological responses	Delayed recognition of vasovagal reactions, blood pressure decline, heart rate abnormalities, and severe pain responses	Multimodal physiological monitoring and abnormal pattern recognition	ECG, PPG, HRV, continuous blood pressure monitoring, electrodermal activity, machine learning	Enables early detection of abnormal autonomic responses before overt symptoms and supports timely intervention	Research from related syncope and autonomic monitoring fields	Current evidence mainly derives from tilt-table testing and syncope populations; acupuncture stimulation, pain, and anxiety may influence autonomic signals; warning thresholds and specificity remain unclear	(38–40)
Post-procedural	Needle safety verification	Retained needles, missed needle removal, and undetected visible needle abnormalities	Automated needle detection, counting, and status verification	YOLO, Oriented R-CNN, object detection, image recognition	Supports needle counting and status verification before and after needle removal, reducing risks associated with manual checking errors	Technical validation studies in acupuncture-related applications	Missed detection may occur with small, reflective, overlapping, or occluded needles; conventional imaging cannot reliably detect internal needle fragments; reduction of real-world retained needle events remains unproven	(11, 41)
	Active adverse event surveillance	Underreporting of mild, delayed, or out-of-clinic adverse events	Digital follow-up and adverse event text identification	NLP, mobile health, patient-reported outcomes, LLM-assisted text analysis	Extends the safety monitoring window and improves detection of mild and delayed adverse events	AI research from related healthcare fields	Patient descriptions lack standardized terminology; models can generally only identify suspected events and cannot independently establish causality with acupuncture; LLMs face challenges related to hallucination, privacy, and accountability	(42–44)
	Safety data management and continuous quality improvement	Fragmented information on patient risk factors, acupuncture parameters, and adverse events prevents effective feedback and optimization	Structured safety databases and intelligent quality analytics	Registries, data standardization, NLP, real-world data analysis, machine learning	Establishes associations among patient characteristics, procedural parameters, and safety outcomes to support risk identification, event surveillance, and workflow optimization	Real-world acupuncture studies combined with healthcare AI research	Lack of acupuncture-specific databases integrating patient characteristics, acupoints, procedural parameters, and follow-up outcomes; inconsistent definitions, severity grading systems, and causality assessment criteria across institutions	(18–20, 42–44)

### 3.1 Pre-procedural stage: from standardized procedures toward individualized safety boundaries

The pre-procedural stage represents the first line of defense in acupuncture patient safety management, with the primary goals of identifying potential risks and developing individualized treatment strategies. Conventional assessment mainly relies on medical history collection, contraindication screening, and practitioner experience. However, substantial variations exist among patients in terms of underlying health conditions, local anatomy, and appropriate procedural parameters, meaning that identical acupoints and acupuncture protocols do not necessarily correspond to equivalent safety boundaries across individuals. Therefore, AI applications before acupuncture have evolved beyond simple acupoint localization toward integrated risk assessment, anatomical evaluation, and needle trajectory planning. The ultimate goal is to incorporate patient-specific and anatomical information to establish more precise safety boundaries before treatment initiation (Table 1).

Current research demonstrates a transition from superficial landmark-based localization toward deeper anatomical safety assessment. Real-world acupuncture studies have identified risk factors associated with adverse events such as bleeding, pneumothorax, and nerve injury, providing an important data foundation for AI-assisted risk prediction. Meanwhile, computer vision, medical imaging, and augmented reality technologies have improved acupoint recognition and anatomical visualization, facilitating a shift from conventional surface-based localization toward individualized planning based on underlying anatomical structures. Thus, the value of AI in the pre-procedural stage extends beyond simply “locating acupoints”; rather, it enables the integration of patient risk profiles, local anatomical characteristics, and needle trajectories into a unified decision-making framework.

However, current evidence remains largely limited to algorithm development, anatomical investigations, and prototype validation, with insufficient clinical studies directly demonstrating improvements in patient safety outcomes. Risk prediction models are constrained by the low incidence of severe adverse events, incomplete documentation of critical procedural parameters, and inconsistent outcome definitions. Similarly, studies evaluating acupoint recognition and anatomical assessment are often based on single-center datasets or controlled experimental environments, and their generalizability across diverse body types, ages, postures, and complex clinical settings remains uncertain. More importantly, existing studies primarily assess localization accuracy or system performance rather than whether these technologies actually reduce adverse events. Therefore, future research should move beyond technical validation and prioritize clinical evaluation frameworks centered on patient safety outcomes.

### 3.2 Intra-procedural stage: from experience-based practice toward intelligent decision support

The acupuncture procedure itself represents the stage with the highest concentration of safety risks and the greatest potential for real-time AI-assisted intervention. Unlike the pre-procedural stage, which primarily focuses on risk assessment and treatment planning, intra-procedural AI applications emphasize dynamic monitoring, parameter quantification, and abnormality detection. The central objective is to enhance the visualization, quantification, and traceability of acupuncture procedures while maintaining clinicians as the primary decision-makers (Table 1).

Emerging technologies, including computer vision, ultrasound guidance, intelligent sensing, and robotics, are expanding the potential for objective assessment of acupuncture procedures. Real-time acupoint tracking systems may identify positional deviations caused by changes in patient posture or soft tissue deformation. Ultrasound-guided approaches can visualize the spatial relationship between the needle and critical anatomical structures, including nerves, blood vessels, and the pleura. Force sensors, motion capture systems, and robotic technologies enable the recording of procedural parameters that have traditionally depended on tactile perception and practitioner experience. Multimodal physiological monitoring approaches are also being explored to detect abnormal autonomic responses before clinically apparent symptoms occur. Although these technologies target different risk points, they share a common goal: reducing information gaps and minimizing human-related errors during acupuncture procedures.

Nevertheless, current evidence remains largely derived from engineering validation, small-scale studies, and research from related medical fields. Real-time recognition performance may be affected by patient movement, soft tissue deformation, and operator-related occlusion. The small size of acupuncture needles also presents challenges for ultrasound visualization and accurate needle-tip tracking. Although robotic systems and sensing technologies improve the measurability of procedural parameters, they remain unable to fully reproduce practitioners’ integrated interpretation of tissue resistance, needle sensation, and immediate patient responses. Furthermore, most existing studies continue to use technical metrics, such as recognition rates, localization errors, or control accuracy, as primary endpoints, and evidence demonstrating reductions in real-world clinical adverse events remains lacking. Therefore, future development of intra-procedural AI should shift from purely optimizing algorithmic performance toward human–AI collaborative validation in real-world clinical environments and evaluation based on patient safety outcomes.

### 3.3 Post-procedural stage: from event documentation toward continuous quality improvement

Patient safety in acupuncture does not end when needle removal is completed. Post-procedural needle verification, adverse event surveillance, and quality feedback are equally important components of safety management. However, conventional approaches rely primarily on manual verification and patient-initiated reporting, making mild, delayed, or out-of-clinic events susceptible to underrecognition. Therefore, the primary value of post-procedural AI lies in extending the monitoring window and transforming safety management from isolated event documentation toward continuous data-driven improvement (Table 1).

Automated needle detection, adverse event identification from clinical text, and safety database development represent the major current directions in this field. Automated needle counting and status verification are among the most directly relevant applications for patient safety, with the potential to reduce missed manual checks. Natural language processing and large language models may facilitate the identification of mild or delayed adverse events from electronic health records, patient-reported outcomes, and follow-up documentation. Meanwhile, structured safety databases integrating patient characteristics, acupuncture parameters, and safety outcomes may provide essential foundations for risk identification, trend monitoring, and workflow optimization. Accordingly, the role of post-procedural AI is evolving from merely detecting adverse events toward supporting continuous quality improvement.

However, this field remains at an early stage of development. Needle detection systems may still experience missed detections under complex conditions involving occlusion, reflections, or overlapping needles. Although text-based AI models can improve the efficiency of potential adverse event screening, they cannot independently determine causal relationships between events and acupuncture procedures and face challenges related to privacy protection, hallucination risks, and reliability. In addition, acupuncture currently lacks standardized, integrated databases incorporating patient characteristics, acupoint information, procedural parameters, adverse events, and long-term outcomes. Variability among institutions in adverse event definitions, severity grading, and causality assessment further limits data integration and model development. Therefore, future efforts should move beyond isolated event detection toward the establishment of standardized data infrastructures and continuous learning systems, providing the foundation for comprehensive intelligent patient safety management across the entire acupuncture care pathway.

## 4 Challenges and future perspectives

### 4.1 Current challenges

In recent years, the application of AI in acupuncture has advanced rapidly, with emerging research spanning acupoint localization, imaging-guided navigation, needle monitoring, and intelligent device development. However, the current evidence base remains largely focused on algorithm development, prototype validation, and small-scale technical evaluations. Clinical studies that directly assess patient safety outcomes as primary endpoints remain scarce. Most existing investigations emphasize technical metrics, such as acupoint recognition accuracy, object detection performance, or image segmentation precision, while relatively few evaluate whether these technologies can effectively reduce clinically relevant adverse events, including pneumothorax, neurovascular injury, or retained needles. Therefore, current applications of AI in acupuncture safety primarily demonstrate technical feasibility rather than sufficient evidence of clinical benefit. Future research should prioritize multicenter, prospective clinical studies that incorporate meaningful safety outcomes, including adverse event rates, risk prediction performance, early warning accuracy, and improvements in clinical workflow efficiency, to further validate the real-world value of AI technologies.

Moreover, the highly individualized and experience-dependent nature of acupuncture practice presents substantial challenges for clinical translation of AI models. Variations exist among different regions, acupuncture traditions, and healthcare institutions regarding acupoint localization methods, needling techniques, and procedural workflows. Meanwhile, heterogeneity in patient age, body habitus, local anatomical structures, and underlying diseases further complicates the development of universally applicable models. Currently, most AI systems are developed using single-center datasets, limited sample sizes, or highly standardized experimental data, with insufficient external validation across institutions, populations, and real-world clinical environments. In addition, the acupuncture field has not yet established unified standards for data acquisition, adverse event classification, or safety evaluation. The lack of standardized frameworks limits data sharing and integration across studies, thereby restricting model optimization, scalability, and generalizability.

Beyond technical limitations, the clinical implementation of AI-based acupuncture safety management involves broader challenges related to algorithm interpretability, human–AI collaboration, data privacy, accountability, and regulatory approval. Acupuncture practice depends not only on anatomical localization but also on dynamic adjustments based on patient responses, changes in needle sensation, and practitioner expertise. Therefore, AI should be positioned primarily as a decision-support tool rather than a substitute for autonomous clinical judgment or procedural execution. Establishing safe, trustworthy, and standardized human–AI collaborative frameworks, together with appropriate ethical governance and regulatory pathways, will be essential for translating AI technologies into routine acupuncture safety management.

### 4.2 Future perspectives

With the continued advancement of AI, multimodal medical imaging, intelligent sensing technologies, and digital health platforms, acupuncture safety management is expected to gradually evolve from experience-driven practice toward data-driven and intelligent decision support. Future research should prioritize multicenter, high-quality clinical investigations and establish standardized databases encompassing patient characteristics, local anatomical features, acupuncture parameters, adverse events, and long-term follow-up outcomes. Such comprehensive data infrastructures will provide essential foundations for AI model development, external validation, and continuous optimization. Meanwhile, further efforts are needed to refine the definitions, classification systems, and evaluation criteria for acupuncture-related safety events, thereby improving data consistency and comparability across studies and facilitating the accumulation of real-world evidence and evidence-based assessment.

Future progress will require the integrated application of multiple technologies, including computer vision, medical imaging, intelligent sensing, wearable devices, large language models, and robotics. These approaches may collectively enable seamless integration of pre-treatment risk assessment, intra-procedural real-time monitoring, and post-treatment continuous surveillance, ultimately establishing an intelligent safety management framework across the entire acupuncture care pathway. At the same time, greater emphasis should be placed on developing effective human–AI collaborative models. Rather than pursuing complete procedural automation, AI should be designed to augment clinicians’ expertise and preserve their central role in clinical decision-making while providing intelligent support for risk identification, precision intervention, quality control, and continuous improvement. Such a balanced approach will be essential for achieving safe, trustworthy, and clinically meaningful integration of AI into acupuncture practice.
